# Microbial Anti-Inflammatory Molecule (MAM) from *Faecalibacterium prausnitzii* Shows a Protective Effect on DNBS and DSS-Induced Colitis Model in Mice through Inhibition of NF-κB Pathway

**DOI:** 10.3389/fmicb.2017.00114

**Published:** 2017-02-01

**Authors:** Natalia M. Breyner, Cristophe Michon, Cassiana S. de Sousa, Priscilla B. Vilas Boas, Florian Chain, Vasco A. Azevedo, Philippe Langella, Jean M. Chatel

**Affiliations:** ^1^Micalis Institute, Institut National de la Recherche Agronomique (INRA), AgroParisTech, Université Paris-SaclayJouy-en-Josas, France; ^2^Laboratorio de Genetica Celular e Molecular, Departamento de Microbiologia, Universidade Federal de Minas GeraisBelo Horizonte, Brazil

**Keywords:** *Faecalibacterium prausnitzii*, inflammation, colitis models, MAM (Microbial Anti-inflammatory Molecule), *Lactococcus lactis*, NF-κB

## Abstract

*Faecalibacterium prausnitzii* and its supernatant showed protective effects in different chemically-induced colitis models in mice. Recently, we described 7 peptides found in the *F. prausnitzii* supernatant, all belonging to a protein called Microbial Anti-inflammatory Molecule (MAM). These peptides were able to inhibit NF-κB pathway *in vitro* and showed anti-inflammatory properties *in vivo* in a DiNitroBenzene Sulfate (DNBS)-induced colitis model. In this current proof we tested MAM effect on NF-κB pathway *in vivo*, using a transgenic model of mice producing luciferase under the control of NF-κB promoter. Moreover, we tested this protein on Dextran Sodium Sulfate (DSS)-induced colitis in mice. To study the effect of MAM we orally administered to the mice a *Lactococcus lactis* strain carrying a plasmid containing the cDNA of MAM under the control of a eukaryotic promoter. *L. lactis* delivered plasmids in epithelial cells of the intestinal membrane allowing thus the production of MAM directly by host. We showed that MAM administration inhibits NF-κB pathway *in vivo*. We confirmed the anti-inflammatory properties of MAM in DNBS-induced colitis but also in DSS model. In DSS model MAM was able to inhibit Th1 and Th17 immune response while in DNBS model MAM reduced Th1, Th2, and Th17 immune response and increased TGFβ production.

## Introduction

Intestinal microbiota homeostasis contributes to the protective mechanism of intestinal mucosa against the development of chronic inflammation. In mice, some commensal bacteria such as segmented filamentous bacteria (Gaboriau-Routhiau et al., 2009; Ivanov et al., 2009), *Bacteroides fragilis* (Round and Mazmanian, 2017) and Clostridia members as *Faecalibacterium prausnitzii* are able to shape gut immune responses (Sokol et al., 2008; Atarashi et al., 2013). The *F. prausnitzii* bacterium accounts for 3–5% of total fecal bacteria and is one of the predominant bacterial groups in human feces. Decreased gut levels of *F. prausnitzii* can result in reduced capacity of self-defense against inflammatory reactions. This protective mechanism may involve the inhibition of pro-inflammatory cytokines and stimulation of anti-inflammatory cytokines secretion by active molecules (Zhang et al., 2014; Quévrain et al., 2016a). One of the mechanisms used by *F. prausnitzii* to inhibit the inflammation is the secretion of bioactive molecules that are able to block nuclear factor kB (NF-κB) activation (Sokol et al., 2008). We recently demonstrated the NF-κB blockage by a bioactive molecule, named Microbial Anti-inflammatory Molecule (MAM), from *F. prausnitzii* in epithelial cells culture (Quévrain et al., 2016a). Years of studies demonstrated some others bioactive molecules able to reduce intestinal inflammation. For example, there are two proteins secreted by *Lactobacillus rhamnosus* GG, p75, and p40, which are able to inhibit epithelial cells apoptosis induced by pro-inflammatory cytokines (Yan et al., 2007). In other hand, one of the best characterized bioactive molecule produced by commensal bacteria is Polysaccharide A (PSA) from *B. fragilis*, a commensal bacterium exhibiting anti-inflammatory properties (Mazmanian et al., 2005, 2008). PSA, which can be found at the surface of vesicles secreted by *B. fragilis*, induce the conversion of CD4 (+) T cells into Foxp3 (+) Treg cells reducing thus the intestinal inflammation (Round and Mazmanian, 2017). These findings highlighted that the searching for such bioactive molecules remains challenging scientifically but could open the door to innovative therapeutic strategies.

Since we described that the loss of *F. prausnitzii* is predictive of Crohn's Disease (CD) relapse after surgery (Sokol et al., 2008) lots of progresses have been made in the comprehension of *F. prausnitzii* role and mechanisms of action (Miquel et al., 2013). Inflammatory Bowel Diseases (IBD), CD and Ulcerative Colitis (UC), remains big issues for public health because only suspensive treatment are available. *F. prausnitzii* and its supernatant showed protective effects in various chemically-induced colitis models in mice (Sokol et al., 2008; Martín et al., 2014, 2015). Recently, we showed that MAM has anti-inflammatory properties *in vivo* in a DNBS-induced colitis model (Quévrain et al., 2016a). In order to do so, we used a food grade bacterium, *Lactococcus lactis*, modified to contain a plasmid with an expression cassette carrying the cDNA coding for MAM under the control of a eukaryotic promoter (pCMV). We have demonstrated that such recombinant *L. lactis* strains are able to transfer fully functional plasmids to eukaryotic cells *in vitro* and *in vivo* resulting in production of protein of interest by the host (Chatel et al., 2008; Del Carmen et al., 2013; Aubry et al., 2015; Souza et al., 2016). *In vitro*, the percentage of cells expressing GFP after co-incubation with *L. lactis* carrying GFP cDNA could reach 1% (Innocentin et al., 2009). As described with MAM or GFP, plasmid transfer occurred in small intestine but also in colon (Almeida et al., 2014; Quévrain et al., 2016a). The number of enterocytes transfected was two times more in colon than small intestine (Almeida et al., 2014) but we have shown recently that plasmid transfer targeted also 5% of the DCs in small intestine or colon (Michon et al., 2015).

Here we wanted to go further in the description of the anti-inflammatory properties of MAM. In order to do so, we used a transgenic mice model where luciferase expression is under the control of NF-κB promoter to test the inhibitory effect of MAM on NF-κB pathway *in vivo*. We also characterized the effect of MAM on DSS-induced colitis model.

## Materials and methods

### Bacterial strains and growth conditions

*Lactococcus lactis* MG1363 containing pILEMPTY (LL-pILEMPTY) plasmid and *L. lactis* MG1363 containing pILMAM plasmid (LL-pILMAM) (Quévrain et al., 2016a) were grown in M17 medium (Difco) supplemented with 1% glucose and erythromycin (10 μg/mL) at 30°C without agitation overnight. The next day, the cultures were diluted 1/20 in M17 medium and grown up at 30°C without agitation. Based in our knowledge, at OD = 1 the bacteria concentration is around 5 × 10^8^ CFU/mL. Mice were gavaged with 5 × 10^9^ CFU/mouse. For all gavages, aliquots 1OX concentrate were previously prepared as described and frozen at −80°C. To use, aliquots were gradually taw on ice bath to preserve all structures and diluted with PBS.

### Mice experiment

This study was carried out in accordance with the guidelines of the local ethics committee. Housing conditions and procedures were specified by the French Law regarding the protection of laboratory animals (authorization #78–149 of the French Veterinary Services). Sets of 6-week-old C57BL/6J mice (*n* = 8 per group) (Janvier, France) were housed in groups of 4 mice per plastic cage, under standard environmental conditions with free access to food and water in the Animal Facility of National Institute of Agronomic Research.

#### DNBS-induced colitis

Colitis was induced in mice under light anesthesia by a single intra-rectal instillation of DNBS (100 mg. kg^−1^ of body weight) diluted in 30% ethanol. During all long experiment, from 7 days before the DNBS (MPBio) instillation to the sacrifice of the mice, mice were fed daily by intragastric gavage with recombinant strains (LL-pILEMPTY and LL-pILMAM 5 × 10^9^ CFU/200 μL/mouse) or with PBS.

Mice were monitored daily for weight loss. Mice were sacrificed at day 4, the abdomen was opened by midline incision and the descending colon and Mesenteric Lymphatic Node (MLN) were removed. MLN were stocked in RPMI medium on ice and after mashed and cells counting to stimulate with anti-CD3 and anti-CD28 for 48 h in 37°C and 5% CO_2_. Colon was rinsed in PBS, opened along the anti-mesenteric border, cleaned and cut for cytokines assays.

#### DSS-induced colitis

Colitis was induced in mice by oral administration of 2.5% (w/v) of Dextran Sulfate Sodium Salt (DSS) at 36,000–50,000 of molecular weight (MPBio) dissolved in drinking water from day 0 to day 7. During all long experiment, from 7 days before the DSS administration to the sacrifice day (D14), mice were fed by intragastric gavage with recombinant strains (LL-pILEMPTY and LL-pILMAM–5 × 10^9^ CFU/200 μL/mouse) or with PBS.

Mice were monitored daily for weight loss, stool consistency, and fecal occult blood (Hemoccult, Beckman Coulter). Disease Activity Index (DAI) has been calculated according to the protocol by Cooper et al. (1993). The mice were sacrificed at day 14 and MLN and colon were collected as described above.

### Protein extraction in colon

One centimeter of colonic tissue was mashed by GentleMax™ (Miltenyl Biotec) in 1 mL of PBS plus anti-protease (Roche). The lysate was centrifuged and the supernatant as used to measure cytokine level by ELISA (Mabtech). The cytokines tested were Th1- related cytokine (IFNγ and IL12); Th2-related cytokines (IL4 and IL5); Th17-related cytokine (IL17) and Treg–related cytokines (IL10 and TGFβ), Th22- related cytokine (IL22) and IL6 (NF-κB pathway).

### Interleukin secretion by stimulated lymphocytes

Mesenteric Lymph Nodes (MLN) isolated from mice were mashed and filtered (70 μm, BD biosciences). Lymphocytes were counted by flow cytometry (Accuri C6) and suspended in RPMI (Lonza) with 100 Unit of Streptomycin, Penicillin, PAA Laboratories and 10% Fetal Calf Serum (FCS) (Lonza) at a concentration of 2,5.10^6^ cells/mL in 24 wells plate (Costar) pre-incubated with anti-CD3 and anti-CD28 antibodies, 4 μg/mL of each antibody (eBioscience) in PBS with 0.5% FCS. Plates were incubated 48 h at 37°C, 5% of CO_2_ and cytokine level was assessed by ELISA (Mabtech). The cytokines tested were Th1- related cytokine (IFNγ); Th2-related cytokines (IL5); Th17-related cytokine (IL17) and Treg–related cytokines (IL10 and TGFβ) and Th22- related cytokine (IL22).

### IVIS (analysis *In vivo*)

NF-κB-luciferase mice, transgenic mice model where luciferase expression is under the control of NF-κB promoter, were used to test the effect of MAM on NF-κB pathway *in vivo*. Mice were anesthetized with a cocktail of 0.1% ketamine (Imalgene 1000, Merial, France) and 0.06% xylazine (Rompun, Alcyon, France) and luciferine 15 mg/mL were administrated by intraperitoneal injection (50 μl). The luminescence of NF-κB recombinant mice was evaluated by IVIS (*In vivo* Imaging System) 200 (Perkin Elmer) at D2 and D4 after DNBS challenge. Region of interest (ROI) measurements were accessed by photon quantification.

### Statistical analysis

All statistics and graphics have been performed on Prism-GraphPad®. Results represent means ± s.e.m. Statistical significance was determined by the Mann-Whitney test. It has been considered that ^*^P < 0.05, ^**^P < 0.01, ^***^P < 0.001.

## Results

### MAM shows a protective effect on DNBS-induced colitis by decreasing NF-κB activation and increasing regulatory and repairing pathways

**Figure 1:**
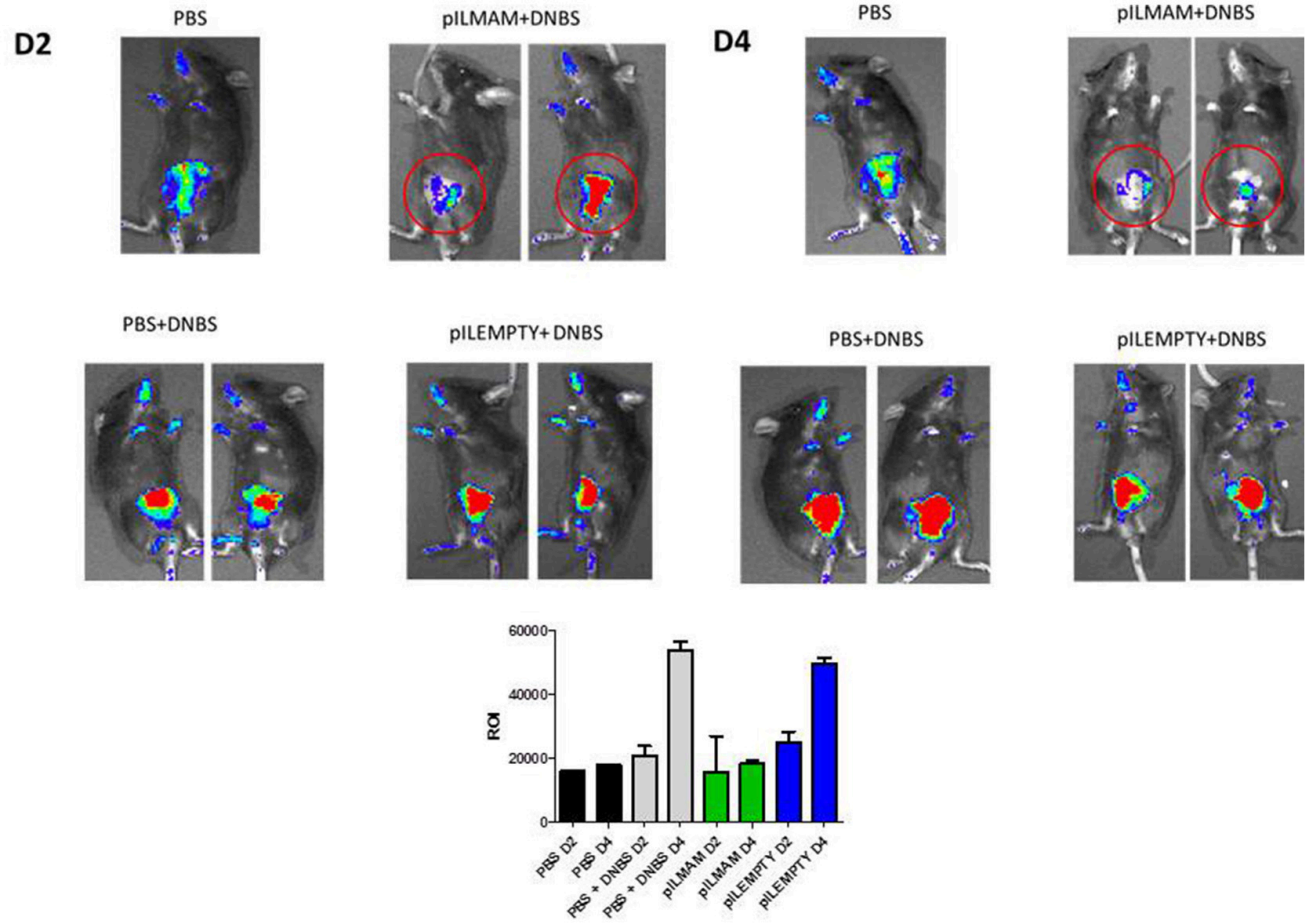
**Inhibition of NF-κB pathway ***in vivo*** after LL-pILMAM administration**. NF-κB luciferase mice, transgenic mice expressing luciferase under the control of NF-κB, were orally administered with PBS, LL-pILEMPTY or LL-pILMAM 7 days before DNBS intrarectal injection (D0) and until sacrifice (D4). NF-κB activation was monitored by luminescence *in vivo* on whole animal using IVIS Spectrum. ROI (Regions of interest) measurements were used to determinate how many photons are radiating from the source.

Colitis was induced in NF-κB-luciferase mice treated or not with LL-pILMAM or LL-pILEMPTY. NF-κB activation was monitored directly on live animals using IVIS. At D4, the luminescence was lower in DNBS- induced colitis mice treated with LL-pILMAM than in LL-pILEMPTY or control mice (PBS). Moreover, the NF-κB signal didn't increase from D2 to D4 in LL-pILMAM treated mice while it increased for LL-pILEMPTY and control mice. This result suggests a protective effect by LL-pILMAM compared with the other groups by inhibiting NF-κB pathway. The region of interest (ROI) measurement confirms these evidences (Figure 1).

**Figure 2:**
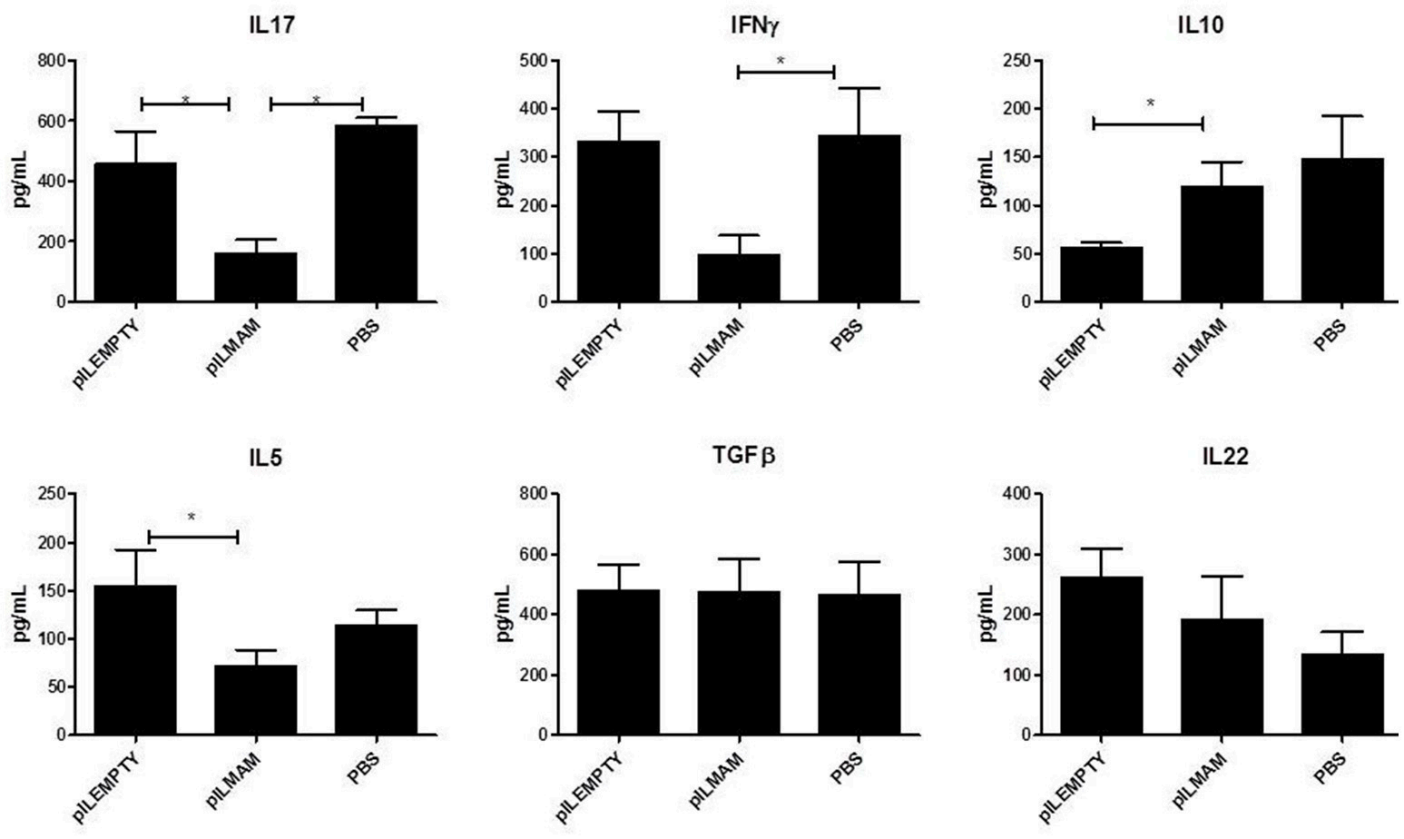
**Cytokines secreted by reactivated lymphocytes from MLN in a DNBS-induced colitis model**. NF-κB luciferase mice were orally administered with PBS, LL-pILEMPTY or LL-pILMAM 7 days before DNBS intrarectal injection (D0) and until sacrifice (D4). At D4 MLN were withdrawn and isolated lymphocytes reactivated by anti-CD3/anti-CD28 antibodies. Cytokine concentration in medium was monitored 48 h after reactivation by ELISA. ^*^P < 0.05.

Pro and anti-inflammatory cytokines secreted by lymphocytes from MLN were monitored 4 days after DNBS administration. As shown in Figure 2, a significant decrease in IL17, IFNγ and IL5 was found in supernatant of lymphocytes from LL-pILMAM treated mice compared to LL-pILEMPTY and control mice. The concentration of IL10 was significantly increased in LL-pILMAM mice compared to LL-pILEMPTY mice, but there is no statistically significant difference compared to control animals. However, no differences in TGFβ and IL22 concentrations were observed among these groups of mice (Figure 2).

**Figure 3:**
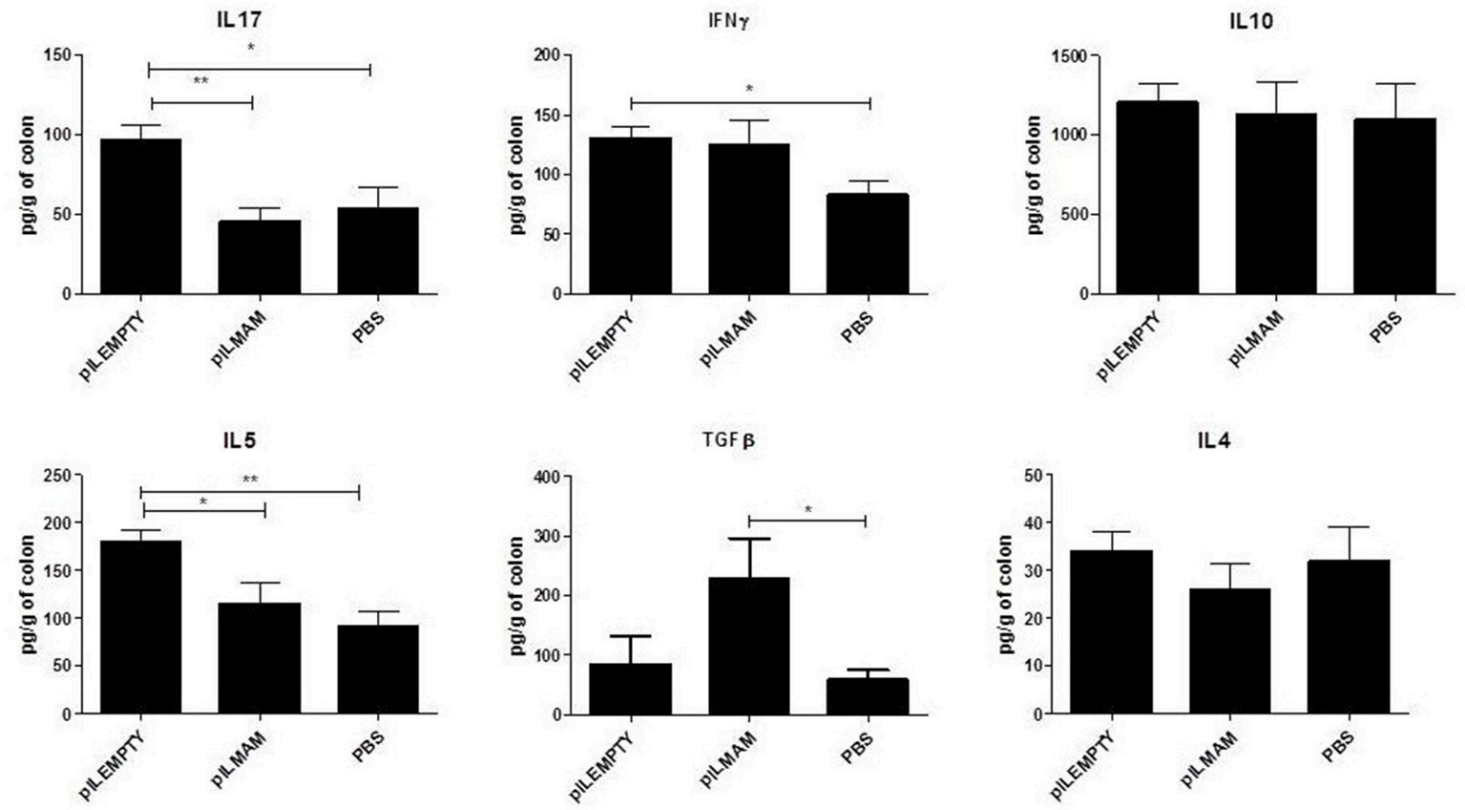
**Cytokines produced by colon tissue in DNBS-induced colitis model**. NF-κB luciferase mice were orally administered with PBS, LL-pILEMPTY or LL-pILMAM 7 days before DNBS intrarectal injection (D0) and until sacrifice (D4). At D4 colon was withdrawn and proteins extracted. Cytokine concentration in protein extracts was monitored by ELISA. ^*^P < 0.05, ^**^P < 0.01.

In parallel, proteins were extracted from colon tissue and cytokines concentrations were assayed. We observed a strong increase of TGFβ in LL-pILMAM treated mice compared to LL-pILEMPTY and control mice. Moreover, the IL5 and IL17 cytokine levels decreased in LL-pILMAM and control mice compared to LL-pILEMPTY mice (Figure 3). We could not observe any differences in IL4 and IL10 production among all groups of mice. It has to be noticed that treatment with LL-pILEMPTY induced an increase of IL5, IL17 and IFNγ compared to control mice.

### Mam protects mice from DSS-induced colitis

**Figure 4:**
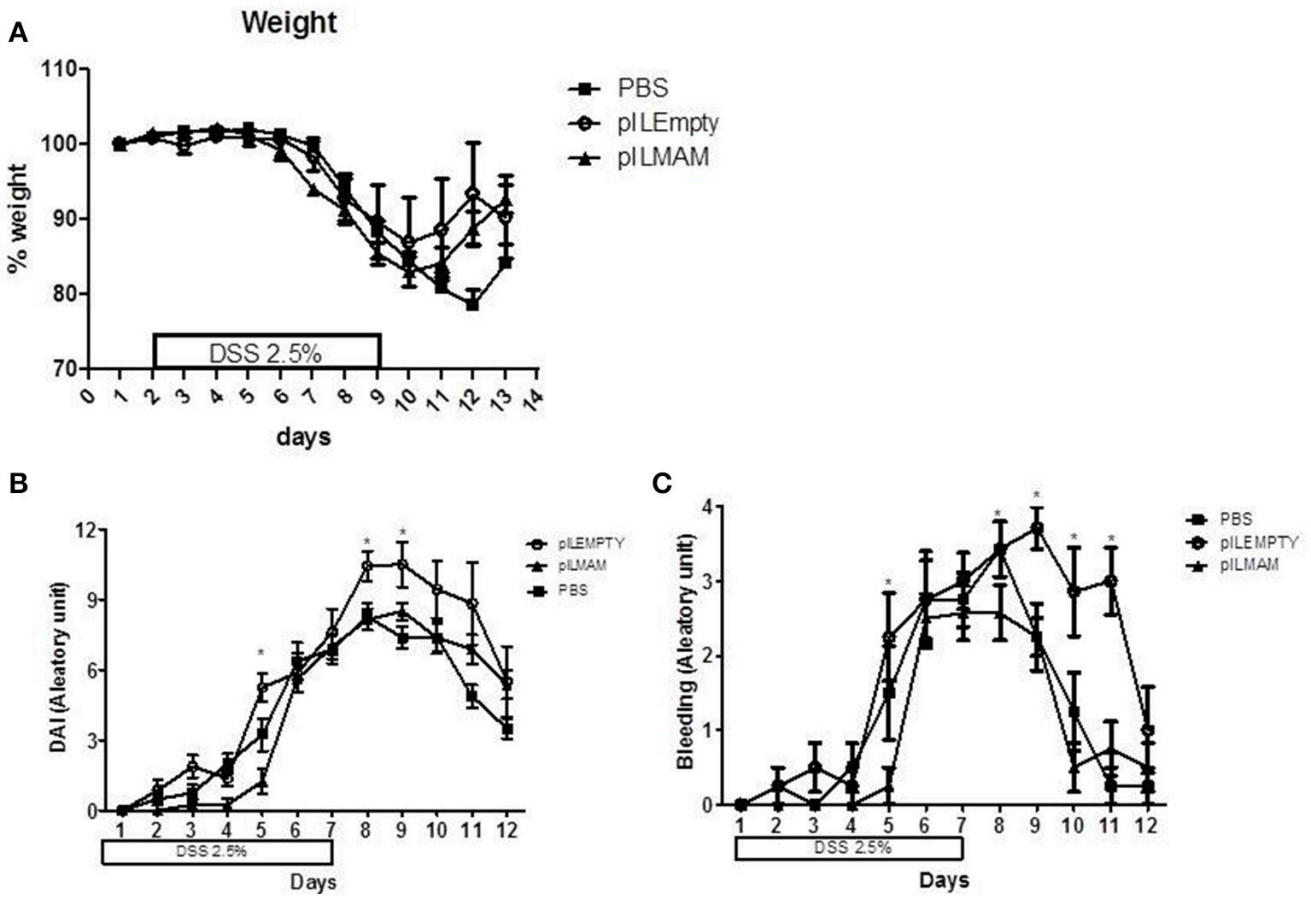
**Effect of MAM on macroscopic scores in a DSS-induced colitis model**. Mice were orally administered with LL-pILMAM, LL-pILEMPTY or PBS 7 days before and during colitis induction. Colitis was induced by adding DSS in drinking water during 7 days (D0-D7). Then DSS was removed from drinking water and mice allowed to recover during 5 days (D7-D12). Weight **(A)** and DAI **(B)** including bleeding **(C)** were monitored daily. ^*^P < 0.05.

To determine the impact of local production of MAM on another chemically-induced colitis model, we performed a DSS-induced colitis model on mice orally administered with LL-pILMAM or LL-pILEMPTY or PBS. Recombinant bacteria were administered 7 days before, during and after colitis induction. We did not observe any difference in the weight loss among these groups of mice (Figure 4A). Oral administration of LL-pILMAM decreased the DAI at D5 compared to pILEMPTY or control mice. At D8 and D9 the DAI difference was only significant compared to pILEMPTY treated mice. At this stage of the colitis daily administration of bacteria seems to increase the DAI (Figure 4B). The effect of MAM is particularly significant on bleeding (Figure 4C).

**Figure 5:**
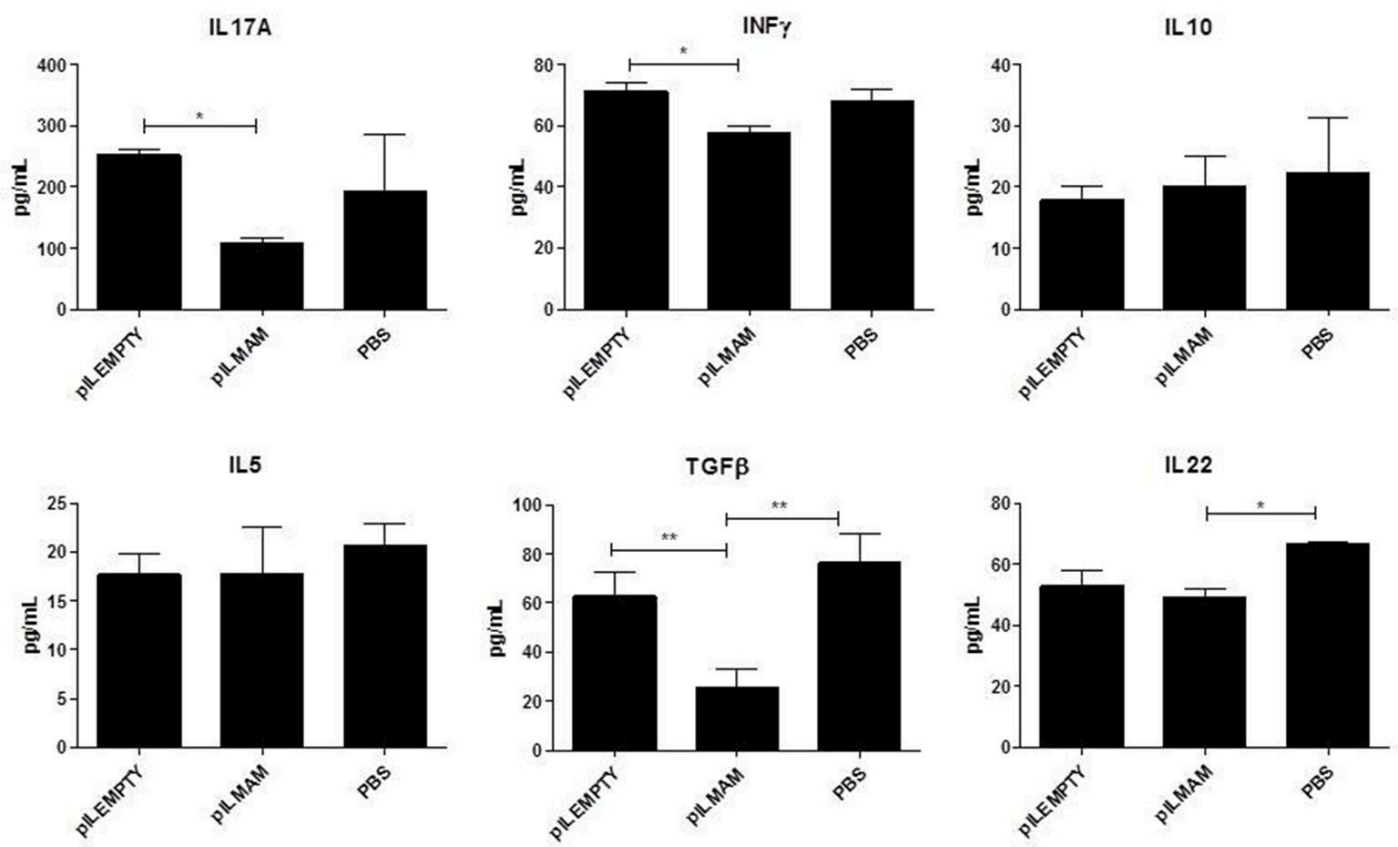
**Cytokines secreted by reactivated lymphocytes from MLN in DSS-induced colitis model**. Mice were orally administered with LL-pILMAM, LL-pILEMPTY or PBS 7 days before and during colitis induction. Colitis was induced by adding DSS in drinking water during 7 days (D0-D7). Then DSS was removed from drinking water and mice allowed to recover during 5 days (D7-D12). At D12 mice were killed, MLN withdrawn, cells were isolated and lymphocytes reactivated with antiCD3/anti-CD28 antibodies. Cytokine concentration in medium was monitored 48 h after reactivation by ELISA. ^*^P < 0.05, ^**^P < 0.01.

**Figure 6:**
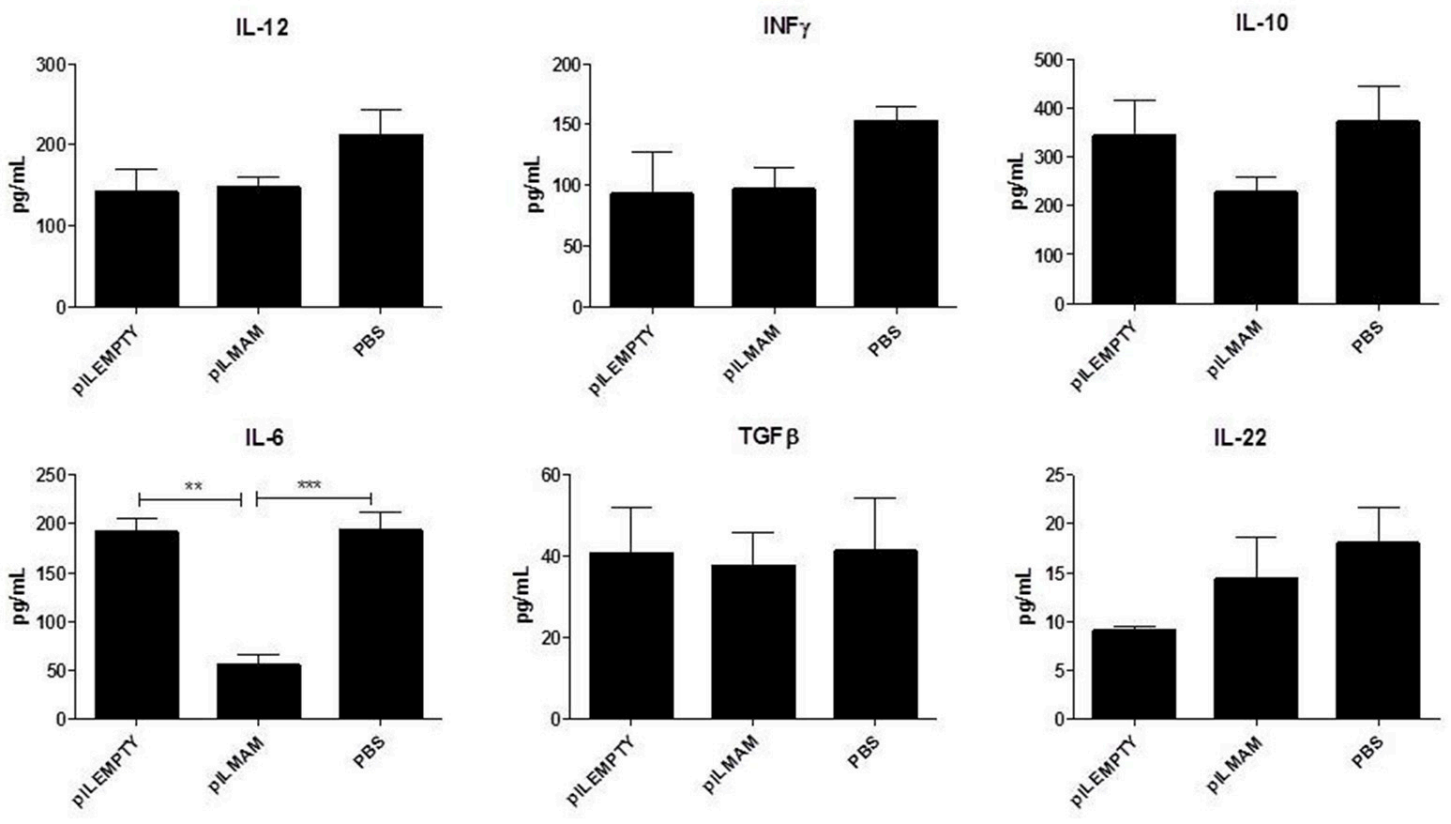
**Cytokines produced by colon tissue in DSS-induced colitis model**. Mice were orally administered with LL-pILMAM, LL-pILEMPTY or PBS 7 days before and during colitis induction. Colitis was induced by adding DSS in drinking water during 7 days (D0-D7). Then DSS was removed from drinking water and mice allowed to recover during 5 days (D7-D12). At D12 mice were killed, colon was withdrawn and proteins extracted. Cytokine concentration in protein extracts was monitored by ELISA. ^**^P < 0.01, ^***^P < 0.001.

After 7 days of inflammation followed by 5 days of recovery, MLN and colon tissues were removed and immune response was analyzed. In MLN supernatant, INFγ and IL17 were both decreased in LL-pILMAM group compared with LL-pILEMPTY (Figure 5) as observed previously in DNBS experiment. TGFβ was also decreased whereas no differences in IL5 and IL10 concentrations were observed among the mice groups. In proteins extracts from colon tissues we could observe only a decrease of IL-6 in LL-pILMAM compared with the other groups (Figure 6). No differences in IFNγ, IL-10, IL-12, TGFβ, and IL-22 concentrations were monitored among the mice groups.

## Discussion

Anti-inflammatory properties of MAM have been characterized by an inhibition of NF-κB *in vitro* and a protective effect in DNBS-induced colitis model on weight loss, macroscopic score and a decrease in IL-17A and IFNγ secreted by activated lymphocytes from MLN (Quévrain et al., 2016a). Here we showed for the first time that MAM inhibits NF-κB pathway *in vivo* by using NF-κB-luciferase transgenic mice. Ours results clearly indicated that delivery of MAM cDNA at intestinal mucosa decreased NF-κB pathway activated by DNBS.

In DSS-induced colitis model, MAM administration didn't modify the weight loss significantly like in DNBS-induced colitis model (Quévrain et al., 2016a) but showed a positive effect on Disease Activity Index (DAI) at several days and especially on bleeding. DAI, which is one of the main macroscopic markers, is established by summing different scores, bleeding, stool consistency, and weight loss reflecting the global physiological state of the digestive tract. These results suggest that MAM is probably able to restore tissue integrity but the mechanism is unclear as IL22, a cytokine involved in tissue repair is slightly decreased. It has to be noticed that this difference is significant considering only pILEMPTY group and not the control PBS group.

We wanted also to characterize more precisely the mechanisms of action of MAM by going deeper in the immune response description and by using a DSS-induced colitis model, both models being commonly used and having different characteristics. Despite the shortcomings, DNBS model is often described to be a good model of CD mainly driven by Th1 and Th17 biased-immune response but also very useful to study the role of adaptive immune response in IBD (Kiesler et al., 2015). One of the other main chemical used to induce colitis models is DSS. DSS model has been described to be closer to UC with a Th2 exacerbated immune response. But DSS inflammation is also mainly driven by innate immune response (Chassaing et al., 2014).

First we confirmed our results by showing that IL-17A (Th17) and IFNγ (Th1) are decreased in MLN from LL-pILMAM group in DNBS-induced colitis. Suppression of Th1 and Th17 cytokine is correlated with beneficial anti-colitis effect (Reyes et al., 2016). We could observe also a decrease of IL-5 and an increase of IL-10 both Th2 cytokines. It has to be noticed that these differences are only significant regarding LL-pILEMPTY group. In DSS model, MAM decreased by 2-fold IL17 secreted by reactivated MLN lymphocytes as in DNBS model. IFNγ is also reduced but less than in DNBS. Surprisingly, TGFβ and IL22 secretion was lower in MAM treated group than in pILEMPTY or PBS group. Thus, MAM has a robust inhibition effect on IL17 and IFNγ secretion in MLN in both models.

We enlarged our investigation field and looked for the cytokines production in colon. In DNBS model, we observed that, like with MLN, IL17, and IL5 are reduced in colon but only regarding pILEMPTY. More striking, we found that TGFβ, which is involved in Treg development, is increased by MAM in colon tissue. In healthy conditions, Treg cells play an important role in controlling immune homeostasis. In IBD, Th1, and Th17 responses overwhelm the control mechanisms of Treg cells. This imbalance in the intestinal immunity of IBD patients, shifting toward the pro-inflammatory side, leads to intestinal inflammation. In DSS model, we observed only a decrease in IL6 production in the colon from mice treated with MAM. Nuclear factor-kB participates in controlling the activation of various pro-inflammatory cytokine genes such as IL6, IFNγ or IL17 suggesting that NF-κB pathway is also turned off in MAM treated mice in DSS colitis model.

As noticed above we observed particularly in DNBS-induced colitis an increase of IL-5, IL-17 and IFNγ in colon tissue of mice treated with LL-pILEMPTY compared to control group and a tendency to decrease IL10 even if the difference is not statistically significant. This profile of response describes *L. lactis* as a pro-inflammatory bacterium. This is in accordance with previous results where we showed that *L. lactis* co-incubated with PBMC give a very low IL10/IL12 ratio (Sokol et al., 2008; Kechaou et al., 2013) or a slight increase of IL-8 secreted by HT-29 after TNFα activation (Kechaou et al., 2013). These two criteria are characteristic of a pro-inflammatory strain. Nevertheless, this slight pro-inflammatory effect of our bacterial vector didn't counterbalance the anti-inflammatory properties of MAM as we have less weight loss or a macroscopic score lower in MAM treated group compared to pILEMPTY treated group (Quévrain et al., 2016a).

Our strategy of plasmid transfer results in MAM expression by epithelial cells. We have no proof that MAM or its peptides produced naturally by *F. prausnitzii* (Quévrain et al., 2016b) have to enter inside the cells of the intestinal membrane to show their protective effect. We tried to produce MAM or its peptides by heterologous protein production or by chemical synthesis without success (Quévrain et al., 2016a). Nevertheless, our strategy of producing MAM by epithelial cell mimics the results obtained with *F. prausnitzii* or its supernatant containing MAM as inhibition of NF-kB, protection against weight loss, increase of IL10 (Sokol et al., 2008), decrease of IL17 (Zhang et al., 2014), decrease of IL6 or IFNγ (Martín et al., 2014) validating our approach.

Our observations suggest that MAM was able to decrease Th1 and Th17 pro-inflammatory cytokines in MLN and colon tissue in both DNBS and DSS colitis model and to enhance TGFβ in DNBS model promoting thus host protective effect and attenuating intestinal inflammation through mechanisms affecting NF-κB activation. Nevertheless, MAM was not able to decrease the Th2 immune response, typically induced in DSS colitis, which results in a less effective protection.

These results can be considered as a new step in the characterization of MAM mechanism of action opening thus the development of innovative therapeutic strategies based on the use of this bioactive molecule. The challenge is to define the immunological regulation associated with this protein or part of this, the location of this protein and the vector used to deliver able to suppress inflammation and determine the optimal means to translate this knowledge to the treatment of inflammatory disease.

## Author contributions

This work was drafted by JC and PL and they received a support financial from INRA transfer to apply in this study. NB and CM were the responsible to perform the experiments, analysis and interpretation of data. CD, PV and FC helped them to develop some key experiments and they participated also in the interpretation of those data. All data were discussed with all authors to establish an integral and coherent analysis. JC, PL, and VA gave the final approval of the version to be published.

### Conflict of interest statement

The authors declare that the research was conducted in the absence of any commercial or financial relationships that could be construed as a potential conflict of interest.
